# SIRT3 protects hepatocytes from oxidative injury by enhancing ROS scavenging and mitochondrial integrity

**DOI:** 10.1038/cddis.2017.564

**Published:** 2017-10-26

**Authors:** Jingxin Liu, Dan Li, Tian Zhang, Qiang Tong, Richard Dequan Ye, Ligen Lin

**Affiliations:** 1 State Key Laboratory of Quality Research in Chinese Medicine, Institute of Chinese Medical Sciences, University of Macau, Avenida da Universidade, Macau, China; 2 Children’s Nutrition Research Center, Baylor College of Medicine, Houston, TX, USA

## Abstract

Evidences of oxidative stress and mitochondrial dysfunction have been recognized in most of clinical and experimental liver diseases. SIRT3, a member of NAD^+^-dependent deacetylases, is mainly localized in mitochondria. So far, the role of SIRT3 in protecting hepatocytes against oxidative stress remains elusive. Herein, we found SIRT3 protein expression is decreased in *tert*-butyl hydroperoxide (*t*-BHP)-treated AML12 cells *in vitro* and primary hepatocytes from CCl_4_-injured mice *in vivo*. To further verify the role of SIRT3 in protecting hepatocytes from *t*-BHP-induced injury, SIRT3 overexpressed AML12 cell line and primary hepatocytes were generated. SIRT3 overexpressed hepatocytes showed improved cell viability upon *t*-BHP challenge, with less intracellular reactive oxygen species (ROS) accumulation. SIRT3 overexpression reduced superoxide dismutase 2 acetylation level and stimulated nuclear factor erythroid 2-related factor 2 nuclear translocation to enhance anti-oxidative capacity. Moreover, SIRT3 deacetylated peroxisome proliferator-activated receptor *γ* coactivator 1*α* to promote mitochondrial biogenesis, and 8-oxoguanine DNA glycosylase 1 to orchestrate DNA repair, resulting in improved mitochondrial function. Through deacetylating Ku70, SIRT3 also abated mitochondrial translocation of dynamin-related protein 1, to attenuate mitochondrial fragmentation in *t*-BHP-injured hepatocytes. These results suggested that SIRT3 protected hepatocytes against oxidative stress by enhancing ROS scavenging and maintaining mitochondrial integrity.

Evidence of oxidative stress has been recognized in most of clinical and experimental liver diseases, including alcoholic liver disease, hepatitis C virus infection, nonalcoholic fatty liver disease, and genetic hemochromatosis.^1^ During liver damage, reactive oxygen species (ROS) cause hepatocyte dysfunction and apoptosis, infiltration of Kupffer cells into the liver, and activation of hepatic stellate cells.^2^ ROS are mainly generated from the endoplasmic reticulum and the electron transport chain in the mitochondria of hepatocytes. Hepatocytes maintain balance between antioxidants and oxidants under normal conditions. Oxidative stress is a state when the cellular oxidant/antioxidant redox balance is altered in favor of the oxidant state.^3^ Thus, targeting excessive ROS accumulation is an effective method to attenuate oxidative stress-induced liver damage. Mitochondrial dysfunction is often associated with increased ROS production. ROS decrease mitochondrial function by affecting the replication and transcription of mitochondrial DNA, causing enhanced ROS production and further damage to mitochondrial DNA.^2^ Mitochondrial ROS homeostasis is important in preventing oxidative injury in hepatocytes.

Impaired mitochondria definitely link with the onset and perpetuation of liver diseases. Altered mitochondrial functions have indeed been documented in a variety of chronic liver diseases including hepatic steatosis, nonalcoholic fatty liver disease, and viral hepatitis.^4, 5^ Dysfunction of mitochondria indicates loss of mitochondrial integrity including impairment of the electron transport chain, mitochondrial fragmentation, mitochondrial DNA damage. Adequate mitochondrial integrity maintains activity of critical enzymes.^6^

SIRT3, a member of NAD^+^-dependent deacetylases, is mainly localized in mitochondria.^7^ Lysine acetylation is a post-translational modification that regulates mitochondrial enzyme activity, and >20% of mitochondrial proteins are regulated by acetylation at lysine residues.^8^ The SIRT3 protein is transported into the mitochondrial matrix and then activated through proteolytic processing at the N-terminus.^9, 10^ SIRT3 expression is increased in response to fasting, caloric restriction, or exercise.^11, 12^ SIRT3 deacetylates many enzymes involved in response to oxidative stress and mitochondrial integrity. Peroxisome proliferator-activated receptor *γ* coactivator 1*α* (PGC-1*α*) regulates mitochondrial biogenesis, which is critical to the maintenance of energy metabolism and mitochondrial function.^13^ Recently, it was reported that SIRT3 upregulates the expression of PGC-1*α* through deacetylation of FOXO3.^14^ SIRT3 deacetylates superoxide dismutase 2 (SOD2)^15^ and isocitrate dehydrogenase 2^16^ to augment anti-oxidative capacity. Recent studies have disclosed that SIRT3 is involved in promoting mitochondrial biogenesis and correcting mitochondrial structure defects.^14, 17^ Moreover, Ku70 is capable of binding with SIRT3 physically to be deacetylated, which results in impeding of Bax mitochondrial translocation.^18, 19^ However, the role of SIRT3 in protecting hepatocytes against oxidative stress is not fully characterized.

In the present study, we demonstrated oxidative stress depletes SIRT3 expression in liver, and uncovered the role of SIRT3 in enhancing ROS-scavenging through deacetylation of SOD2, and mitochondrial integrity through deacetylation of PGC-1*α* and Ku70.

## Results

### SIRT3 expression is decreased in oxidative injured hepatocytes *in vitro* and *in vivo*

**Figure 1:**
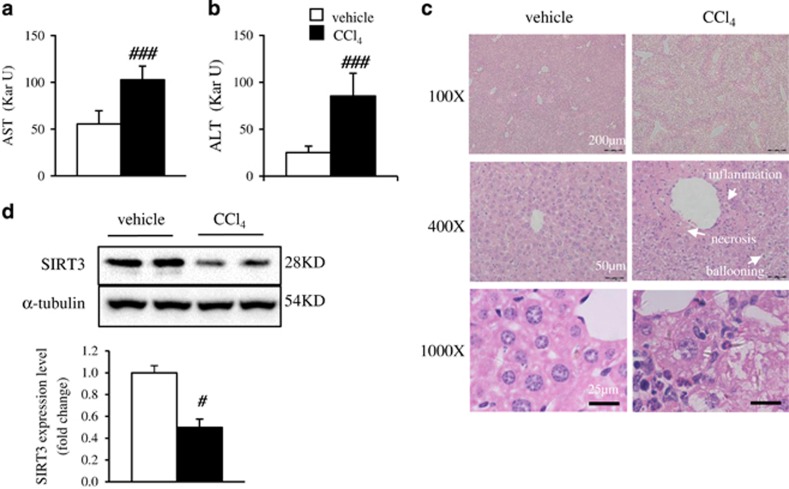
SIRT3 expression is decreased in primary hepatocytes from CCl_4_-induced hepatic damage mice. (**a**) Serum AST levels in mice. (**b**) Serum ALT levels in mice. (**c**) Representative H&E staining of mouse livers. (**d**) SIRT3 protein levels in mouse livers. Data are shown as mean±S.D., *n*=10–12, ^###^*P*<0.001 CCl_4_ *versus* vehicle group

CCl_4_ is known to cause acute liver injury characterized with centrilobular necrosis.^20^ We determined whether SIRT3 expression is changed in primary hepatocytes from CCl_4_-injured mice. In the liver injured mice, aspartate transaminase (AST, Figure 1a) and alanine transaminase (ALT, Figure 1b) levels were significantly increased. The H&E staining was performed to observe the change of liver architecture. More necrosis and inflammation hepatocytes were found in the liver specimens from CCl_4_-treated mice, compared with those of control mice (Figure 1c). As expected, the SIRT3 protein expression was suppressed in the primary hepatocytes from oxidative injured mice (Figure 1d). It suggested SIRT3 might have a key role in protecting hepatocytes from oxidative stress.

**Figure 2:**
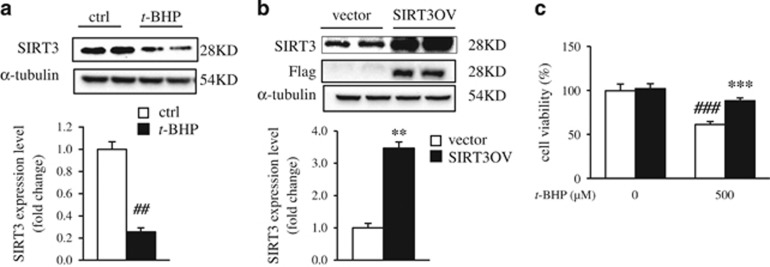
SIRT3 protects AML12 hepatocytes from *t*-BHP-induced oxidative injury. (**a**) SIRT3 protein is decreased in *t*-BHP-treated AML12 cells. (**b**) Generation of SIRT3 overexpressed AML12 cell line. SIRT3 protein levels were detected by western blot analysis and quantified using Image J. (**c**) Cell viability was determined by MTT assay. **P<0.01, and ***P<0.001 SIRTOV *versus* vector cells, and ^##^*P*<0.01 and ^###^*P*<0.001 *t*-BHP-treated *versus* ctrl cells

Consistently, in the murine hepatocyte AML12 cells, the SIRT3 protein expression was also decreased when treated with *tert*-butyl hydroperoxide (*t-*BHP), a widely used oxidative injury inducer (Figure 2a). To directly evaluate the role of SIRT3 in hepatocytes against oxidative stress, we first generated an AML12 cell line expressing a transgene in which the Flag-tag fused to N-terminus of the full-length SIRT3 cDNA (SIRT3OV).^21^ SIRT3OV cells expressed around 3.5-fold more SIRT3 protein than that in the cells transfected with vector plasmid (vector) using western blot analysis (Figure 2b). In addition, the Flag protein was only detected in the SIRT3OV cells (Figure 2b). Interestingly, SIRT3 overexpression exerted profound effect on total protein acetylation levels and apparently downregulated acetylation levels of most protein (Supplementary Figure S1a). Evidence of SIRT3 in protecting hepatocytes from *t*-BHP-induced oxidative injury was evaluated by 3-(4,5-dimethylthiazol-2-yl)-2,5-diphenyltetrazolium bromide (MTT) method. The viability of SIRT3OV cells was ~30% higher than that of the vector cells under 500 *μ*M *t*-BHP treatment, but unchanged under normal condition (Figure 2c), which was further supported by the trypan blue exclusion results (Supplementary Figure S1b). The above data indicated that overexpression SIRT3 significantly protected hepatocytes against oxidative injury.

### SIRT3 enhanced cellular anti-oxidative defense capacity

**Figure 3:**
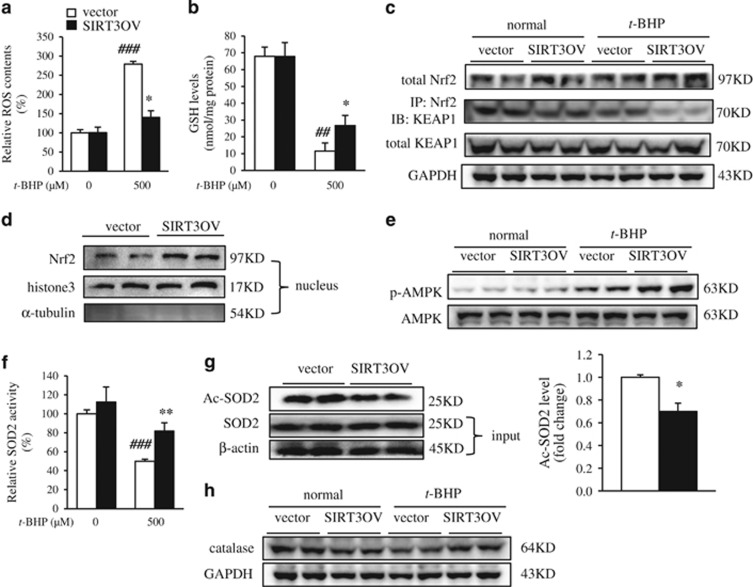
SIRT3 overexpression enhances cellular anti-oxidative defense capacity. (**a**) Intracellular ROS level. (**b**) GSH level. (**c**) Total Nrf2 and Keap1 protein levels. (**d**) Nuclear Nrf2 protein level. (**e**) Phosphorylated and total AMPK protein levels. (**f**) SOD2 activity. (**g**) Acetylated and total SOD2 protein levels. (**h**) Catalase protein level. Data are shown as mean±S.D., *n*=6, *P<0.05 and **P<0.01, vector *versus* SIRT3OV cells, ^##^*P*<0.01, and ^###^*P*<0.001 *t*-BHP-treated *versus* ctrl cells

Prevention of excessive ROS directly relies on antioxidants in cells.^22^ Then, the anti-oxidative capacity of SIRT3OV cells was examined. Using 2,7-dichlorodihydrofluorescein diacetate (DCFH-DA) assay, the intracellular ROS content was significantly boosted in AML12 cells treated with *t*-BHP; and SIRT3 effectively reduced the ROS content in *t*-BHP-injured cells, but not in normal cells (Figure 3a). And the concentration of malondialdehyde, the lipid peroxide product, was also significantly decreased in SIRT3OV cells (Supplementary Figure S2a). GSH is an important non-enzymatic antioxidant, which is capable to prevent ROS-induced damage to important cellular components.^23^ As expected, the GSH level was significantly reduced in the *t*-BHP-treated AML12 cells, which was restored in SIRT3OV cells (Figure 3b). Nuclear factor erythroid 2-related factor 2 (Nrf2) is the master regulator of antioxidant responses, which controls GSH production and regeneration.^24^ Indeed, SIRT3 overexpression remarkably increased the total Nrf2 levels under normal and *t*-BHP-treated conditions (Figure 3c). NRF2 is bound and maintained in cytoplasm by its inhibitor KEAP1 (Kelch-like ECH-associated protein 1), which is released to regulate expression of anti-oxidative genes upon oxidative stress.^25^ Although the total protein did not change, the KEAP1 protein bound to Nrf2 was obviously reduced in the SIRT3OV cells compared with that in the vector cells, under both normal and *t*-BHP-treated conditions (Figure 3c). It suggested that SIRT3 might promote the release of KEAP1 from Nrf2 to stimulate Nrf2 nuclear translocation. Furthermore, the nuclear Nrf2 protein was increased in SIRT3OV cells (Figure 3d). The nuclear translocation of Nrf2 was further confirmed with the immunostaining images (Supplementary Figure S2b). SIRT3 overexpression also increased the phosphorylation level of adenosine 5'-monophosphate (AMP)-activated protein kinase (AMPK) (Figure 3e). SIRT3 was reported to directly deacetylate SOD2 and regulate its activity.^15^ As expected, SOD2 activity was reduced in *t*-BHP-treated AML12 cells, and SIRT3 overexpression markedly restored SOD2 activity (Figure 3f). Although the total SOD2 protein did not change, SIRT3 overexpression markedly reduced the acetylation level of SOD2 in AML12 cells (Figure 3g). Moreover, catalase expression was obviously increased in SIRT3OV cells, especially under *t*-BHP treatment (Figure 3h). These results indicated that SIRT3 enhances ROS-scavenging capability in oxidative injured hepatocytes.

### SIRT3 reversed *t*-BHP-induced loss of mitochondria in hepatocytes

**Figure 4:**
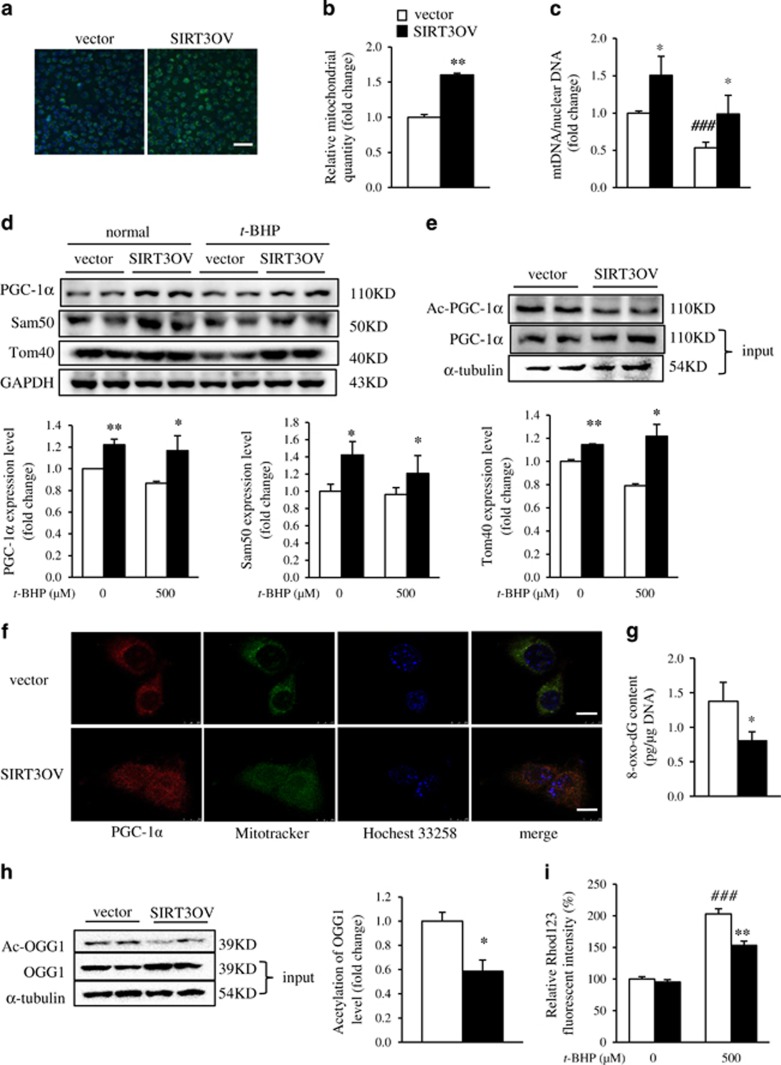
SIRT3 prevents *t*-BHP-induced mitochondrial loss in AML12 hepatocytes. (**a**) Mitochondria were visualized using Mitotracker Green (Green) and nuclei were visualized using DAPI (blue) in an IN Cell Analyzer. Scale bar 100 *μ*m. (**b**) Quantification of mitochondrial quantity by flow cytometry. (**c**) Ratio of mitochondrial DNA/nuclear DNA. (**d**) Protein expressions of PGC-1*α*, Sam50, and Tom40 in vector and SIRT3OV cells, under both normal and induction conditions. (**e**) Acetylated and total PGC-1*α* protein levels. (**f**) Immunofluorescence analyses of PGC-1*α* levels. Cellular PGC-1*α* levels were observed using a Leica TCS SP8 Confocal Laser Scanning Microscope System. Cells were stained with Mitotracker Green (green), PGC-1*α* (red), and Hoechst 33258 (blue). Scale bar, 10 *μ*m. (**g**) Change in 8-oxo-dg level in vector and SIRT3OV cells treated with 500 *μ*M *t*-BHP. (**h**) Acetylated and total OGG1 protein levels. (**i**) Relative Rhod123 fluorescent intensity in vector and SIRT3OV cells, under both normal and induction conditions. Data are shown as mean±S.D., *n*=6, *P<0.0,5 and **P<0.01 vector *versus* SIRT3OV cells, ^###^*P*<0.001 *t*-BHP-treated *versus* ctrl cells

ROS damage is associated with a decline in mitochondrial integrity featured with damage of mitochondrial DNA and proteins.^26^ The Mitotracker Green staining results showed SIRT3 overexpression significantly increased the mitochondrial content in the *t*-BHP-treated hepatocytes (Figure 4a). Furthermore, the flow cytometry results revealed a 50% increase of mitochondrial mass in SIRT3OV cells (Figure 4b), which was further supported by the higher ratio of mtDNA/nuclear DNA in SIRT3OV hepatocytes compared with that in the vector cells (Figure 4c). PGC-1*α* is the master regulator of mitochondrial biogenesis, and its activity is negatively associated with the acetylation level.^27^ Interestingly, SIRT3 overexpression not only upregulated PGC-1*α* protein but also reduced its acetylation level (Figures 4d and e). In addition, the mitochondrial structure proteins, Sam50 and Tom40, were also increased in SIRT3 overexpressed hepatocytes, especially under *t*-BHP treatment (Figure 4d). Decreased acetylation of PGC-1*α* resulted in the nuclear translocation, characterized by the immunostaining images (Figure 4f). Consistently, the mitochondrial transcription factor A, a key participant in mitochondrial genome replication, was significantly increased in SIRT3OV cells (Supplementary Figure S3a). These results suggested that SIRT3 promotes mitochondrial biogenesis in hepatocytes under oxidative stress.

8-Oxoguanine (8-oxo-dG), an oxidized form of guanine, is a marker of DNA oxidative damage. Our result showed 8-oxo-dG level was lower in SIRT3OV cells than that in the vector cells (Figure 4g). 8-Oxoguanine DNA glycosylase (OGG1) is a DNA repair enzyme serving as a deacetylation substrate of SIRT3 in cancer cells.^28^ Western blot analysis showed SIRT3 overexpression increased total OGG1 protein and decreased its acetylation level in oxidative injured hepatocytes (Figure 4h and Supplementary Figure S3b). Mitochondrial membrane potential (ΔΨm) plays a vital role in maintaining the physiological function of mitochondria.^29^ R123 was used as a mitochondrial membrane potential probe because it quenches when entering the mitochondrial membrane in a membrane potential-dependent manner. The results showed *t*-BHP treatment interrupted ΔΨm; SIRT3OV cells possessed higher ΔΨm, indicating improved mitochondrial function (Figure 4i). Taken together, these results suggested that SIRT3 overexpression enhanced mitochondrial content and function in hepatocyte under oxidative stress.

### SIRT3 attenuated mitochondrial fragmentation in oxidative injured hepatocytes

**Figure 5:**
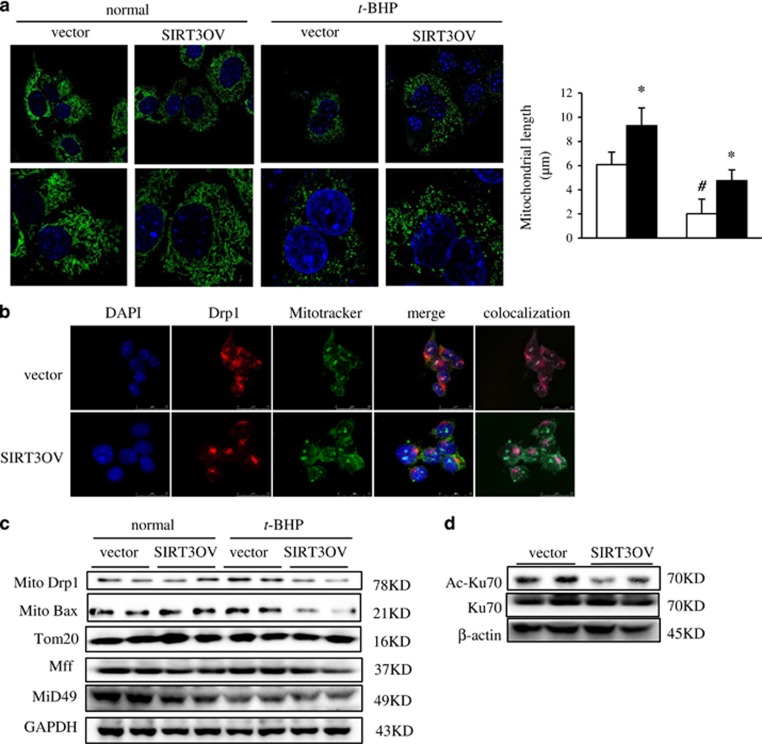
SIRT3 prevents *t*-BHP-induced mitochondria fragmentation in AML12 hepatocytes. (**a**) Representative confocal image of mitochondrial morphology. Nuclei were visualized using Hochest 33258 (blue), and mitochondria were visualized using Mitotracker Green (green). Mitochondrial length was analyzed using Imaris7.4.2 software. After the indicated treatments, mitochondrial were stained with Mitotracker Green in hepatocytes. Z-stack images were analyzed for changes in mitochondrial structure using Imaris7.4.2 software, and 3D reconstructions were generated from z-stack images for analysis of mitochondrial length. (**b**) Immunofluorescence analyses of Drp1 localization. Cells were stained with Mitotracker Green (green), Drp1 (red), and DAPI (blue). Scale bar, 25 *μ*m. (**c**) Drp1 and Bax protein levels in mitochondrial compartment, and Mff and Dip49 protein levels in whole cell lysate. (**d**) Acetylated and total Ku70 protein levels. Data are shown as mean±S.D., *n*=6, *P<0.05 vector *versus* SIRT3OV cells, ^#^*P*<0.05 *t*-BHP-treated *versus* ctrl cells

Mitochondrial morphology is a result of its dynamic changes and abnormal mitochondrial morphology is associated with a variety of disorders and dysfunctions. The balance of fusion and fission events plays a pivotal role in maintaining mitochondrial architecture and function. In the current study, the severe mitochondrial fragmentation was observed in *t*-BHP damaged hepatocytes and SIRT3 overexpression ameliorated the mitochondrial fragmentation (Figure 5a). Drp1 (dynamin-related protein 1) is a conserved GTPase protein that mediates membrane remodeling in mitochondria. Drp1 resides mainly in the cytoplasm with only a small fraction localizing to mitochondria as foci, which is involved in mitochondrial fission.^30^ When activated, Drp1 was recruited to mitochondrial fission sites to process fission which is essential for maintaining mitochondrial number and function. On the contrary, Drp1would lead mitochondrial fragmentation under imbalance of fusion and fission. Thus, we speculated that SIRT3 abated mitochondrial fragmentation mediating through Drp1 recruitment to mitochondria. The immunostaining images showed SIRT3 overexpression significantly inhibited Drp1 mitochondrial recruitment in the *t*-BHP-treated hepatocytes (Figure 5b). Consistently, the western blot analysis showed the mitochondrial Drp1 protein level was reduced in SIRT3OV cells under *t*-BHP treatment, but not in normal cells (Figure 5c). Mff (mitochondrial fission factor) and MiD49 (mitochondrial dynamics protein of 49 KDa) mediate Drp1 mitochondrial translocation.^31^ The results showed the protein expressions of Mff and MiD49 were decreased in SIRT3OV hepatocytes with or without treatment of oxidant (Figure 5c). It was reported that cytosolic Bax targets the mitochondrial outer membrane and co-localizes with Drp1;^32^ and Bax is essential for Drp1-mediated mitochondrial fission.^33^ Ku70, one of the SIRT3 substrates, has capability of hindering the translocation of Bax to mitochondria.^19^ As expected, SIRT3 overexpression dramatically decreased Bax protein in mitochondria from *t*-BHP injured AML12 cells, but not from normal cells (Figure 5c). Although the total Ku70 expression did not change, the acetylated Ku70 was significantly decreased in SIRT3OV hepatocytes (Figure 5d). The above data indicated SIRT3 attenuates mitochondrial fragmentation in oxidative injured hepatocytes mediating through Ku70-Bax-Drp1 axis.

### SIRT3 protects primary hepatocytes from oxidative injury

**Figure 6:**
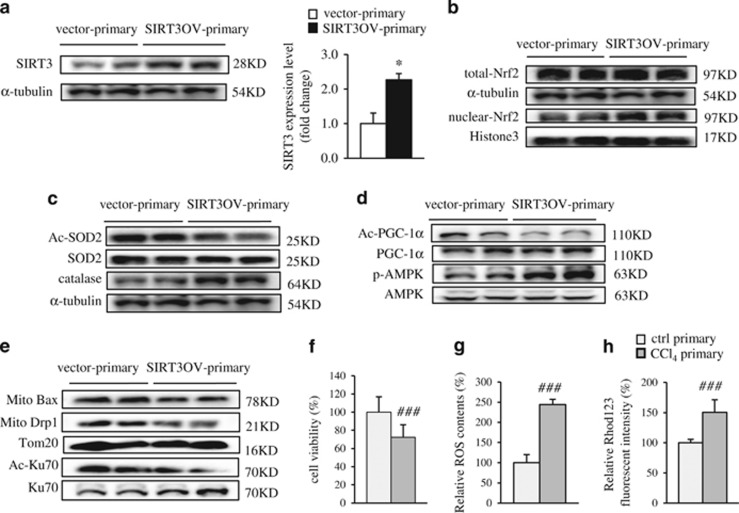
SIRT3 protects primary hepatocytes from oxidative injury. (**a**) Generation of SIRT3 overexpressed primary hepatocytes. SIRT3 protein levels were detected by western blot analysis and quantified using Image J. (**b**) Total and nuclear Nrf2 protein levels. (**c**) SOD2, acetylated SOD2 and catalase protein levels. (**d**) PGC-1*α* and its acetylation levels, and AMPK and p-AMPK protein levels. (**e**) Drp1 and Bax protein levels in mitochondrial compartment, and Ku70 and acetylated Ku70 levels in while cell lysate. Cell viability (**f**), intracellular ROS level (**g**), and relative Rhod123 fluorescent intensity (**h**) were determined in primary hepatocytes from control or CCl_4_-injured mice. Data are shown as mean±S.D., *n*=6, *P<0.05, vector-primary *versus* SIRT3OV-primary cells; ^###^*P*<0.001 CCl_4_ primary *versus* ctrl primary

To confirm the protecting role of SIRT3, we isolated primary hepatocytes from liver of C57BL6 mice, and overexpressed SIRT3 in the primary hepatocytes (SIRT3OV-primary). As shown in Figure 6a, the SIRT3 protein was ~2.3-fold higher in SIRT3OV-primary cells compared with that in the primary hepatocytes expressed vector control (vector-primary). Consistently, SIRT3 overexpression in primary hepatocytes stimulated Nrf2 nuclear translocation (Figure 6b), increased catalase level and reduced SOD2 acetylation level (Figure 6c), to enhance anti-oxidative capacity. Moreover, SIRT3 overexpression activated AMPK and reduced PGC-1*α* acetylation level (Figure 6d), to regulate mitochondrial biogenesis. At last, SIRT3 overexpression decreased Drp1 and Bax proteins in mitochondria, and reduced acetylated Ku70 level, to attenuate mitochondrial fragmentation (Figure 6e). In addition, the primary hepatocytes from CCl_4_-injured mice showed less cell viability (Figure 6f), more intracellular ROS accumulation (Figure 6g) and reduced mitochondrial membrane potential (Figure 6h), when compared with those from control mice. Taken together, overexpression of SIRT3 protects primary hepatocytes from oxidative injury.

### SIRT3 silenced deteriorated oxidative injury in hepatocytes

**Figure 7:**
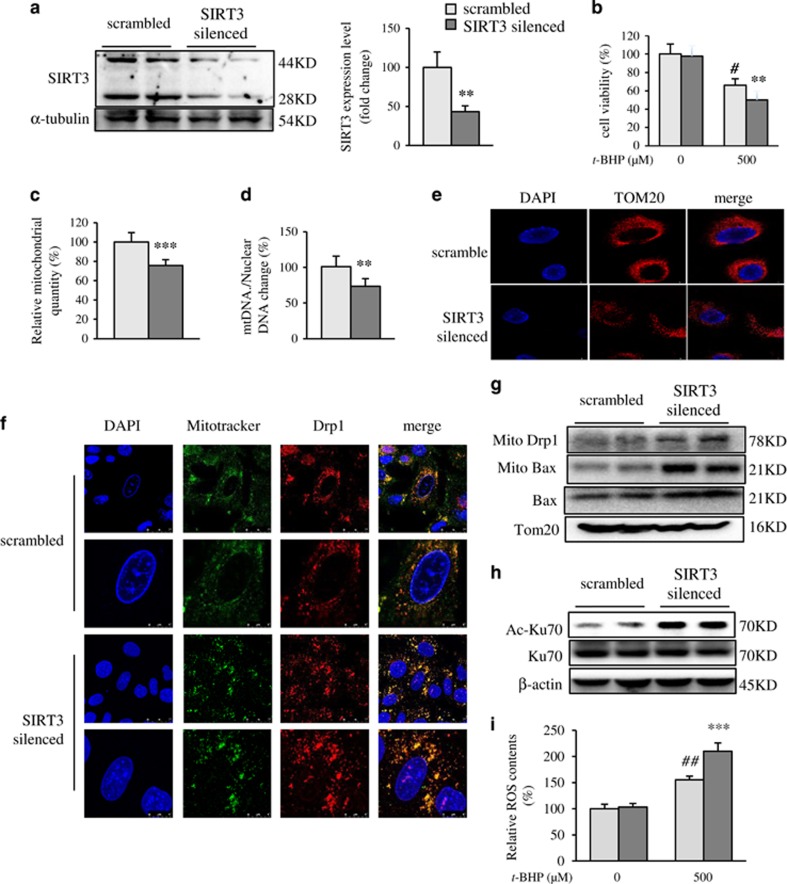
SIRT3 silenced deteriorated oxidative injury in hepatocytes. (**a**) Generation of SIRT3-silenced AML12 cell line expressing shScr or shSIRT3. SIRT3 protein levels in these cells were detected by western blot analysis and quantified using Image J. (**b**) Cell viability was determined by MTT assay before and after treatment of 500 *μ*M *t*-BHP. (**c**) Quantification of mitochondrial content by Mitotracker Green. (**d**) Ratio of mitochondrial DNA/nuclear DNA. (**e**) Representative confocal image of mitochondrial morphology. Nuclei were visualized using Hochest 33258 (blue), and mitochondria were visualized using Tom20 (red). (**f**) Immunofluorescence analyses of Drp1 localization. Cells were stained with Mitotracker Green (green), Drp1 (red), and DAPI (blue). Scale bar, 25 *μ*m. (**g**) Drp1 and Bax protein levels in mitochondrial compartment, and total Bax protein level. (**h**) Acetylated and total Ku70 protein levels. (**i**) Intracellular ROS level. **P<0.01 and ***P<0.001 SIRT3-silenced *versus* scrambled cells, ^#^*P*<0.05 and ^##^*P*<0.01 *t*-BHP-treated *versus* ctrl cells

To further verify the role of SIRT3 in protecting hepatocytes from oxidative injury, a SIRT3-silenced AML12 cell line (SIRT3 silenced) was generated using shRNA-targeting SIRT3. As shown in Figure 7a, SIRT3-silenced cells expressed ~60% less SIRT3 protein compared with that of the cells expressing scrambled shRNA (scrambled). Treatment of *t-*BHP caused more severe cell death in SIRT3-silenced cells compared with that of scrambled cells (Figure 7b). As expected, SIRT3 silenced decreased mitochondrial content, indicating by the flow cytometry analysis of Mitotracker Green (Figure 7c) and the ratio of mtDNA/nuclear DNA (Figure 7d). Consistently, the immunostaining of Tom20 showed a remarkable decrease of mitochondrial quantity in SIRT3-silenced cells (Figure 7e). On the other hand, SIRT3 silenced deteriorated mitochondrial fragmentation in hepatocytes under oxidative stress (Figure 7f). Specifically, the Mander’s coefficient in SIRT3-silenced cells was about threefold higher than that in the scrambled cells using threshold (3.04), suggesting SIRT3 plays an important role in Drp1 mitochondrial translocation. The western blotting results also showed that Drp1 and Bax protein levels were increased markedly in mitochondria from SIRT3-silenced cells (Figure 7g). In addition, the total Bax protein was also increased in SIRT3-silenced cells (Figure 7g). Although the total Ku70 protein didn’t change, the acetylation level of Ku70 was increased remarkably in SIRT3-silenced cells (Figure 7h). Taken together, loss of SIRT3 causes decreased mitochondrial content and severe mitochondrial fragmentation in oxidative injured hepatocytes.

## Discussion

SIRT3 has a vital role in manipulating oxidative stress in various cells and tissues. A previous study showed that SIRT3 expression in hepatocytes decreased in high-fat diet-induced obese mice.^34^ A recent study has suggested that SIRT3 protects oxidative stress mediated by hepatitis B virus X protein expression.^35^ The present results showed that SIRT3 expression is decreased in *t*-BHP-injured hepatocytes *in vitro* and primary hepatocytes from CCl_4_-induced liver damaged mice *in vivo*. Hence, oxidative injury affected SIRT3 expression in hepatocytes, indicating that SIRT3 has a key role in protecting oxidative stress in liver.

Liver contains a large number of mitochondria, which are the predominant source of intracellular ROS. Excessive ROS accumulation results in cell death through the oxidation of polyunsaturated fatty acids in cellular membranes, the substantial number of unprotected protein sulfhydryl groups, and DNA bases.^36^ In hepatocytes, the anti-oxidative system maintains a balance of ROS content, including SOD2, GSH, and catalase. SIRT3 directly deacetylates lysine 122 to regulate SOD2 activity in response to stress.^37^ The current data uncovered SIRT3 decreased the acetylation level of SOD2, but did not change the total protein level, to enhance SOD2 activity, in oxidative injured hepatocytes. Many studies revealed that adequate GSH level offers cellular protection against the noxious effect of intracellular ROS.^38^ The current data showed that SIRT3 overexpression increased GSH level, suggesting improved capacity to attenuate ROS-induced hepatic damage. Nrf2 is a pivotal transcriptional factor capable of recognizing the antioxidant response element through nuclear translocations.^39^ Interestingly, we observed SIRT3 overexpression released the inhibitor KEAP1 from Nrf2 and stimulates Nrf2 nuclear translocation. It is also notable that in some cases Nrf2 induces SIRT3 expression.^40^ Hence, the nuclear translocation of Nrf2 is capable of in turn strengthening SIRT3 function.

Mitochondria integrity is maintained through a series of physical processes such as mitochondrial biogenesis, autophagic of dysfunctional parts, and dynamics control on fission/fusion events. In the present study, the mitochondrial homeostasis was significantly interrupted in the hepatocytes exposed to *t*-BHP, accompanied with decreased mitochondrial content and increased mitochondrial fragmentation. Interestingly, SIRT3 totally reserved mitochondrial integrity in hepatocytes under oxidative stress. In fact, mitochondrial biogenesis acts as a critical aspect for mitochondrial quantity, and SIRT3 was reported to facilitate the process by targeting PGC-1*α* deacetylation.^27, 41, 42^ The current data further supported SIRT3 promoted mitochondrial biogenesis through deacetylating PGC-1*α* in hepatocytes.

AMPK is responsible for monitoring cellular energy status and is regulated through phosphorylation at threonine 172.^43^ AMPK directly stimulates mitochondrial energy production and strengthens mitochondrial biogenesis. AMPK induces PGC-1*α* gene expression and directly phosphorylates PGC-1*α*.^44, 45^ In addition, liver kinase B1, an acetylation substrate of SIRT3, directly phosphorylates and activates AMPK.^12, 46^ The current data showed that SIRT3 overexpression promoted the phosphorylation of AMPK, which could induce PGC-1*α* gene expression, and directly decrease the phosphorylation of PGC-1*α*. Phosphorylation of AMPK is also an underlying factor for Nrf2 nuclear translocation.^47^ In general, SIRT3 stimulates mitochondrial biogenesis in hepatocytes under oxidative stress through the SIRT3-AMPK-PGC-1*α* axis.

SIRT3 may also involve in mitochondrial renewal and hepatocytes proliferation mediating through mitophagy mechanisms. Our preliminary data showed SIRT3 induced autophagy, leading to attenuation of dysfunctional mitochondria mediated cell injury (data not shown). Future studies are required to fully elucidate how SIRT3 regulates mitophagy when responding to oxidative stress.

Mitochondrial integrity, including DNA, protein, and organelle integrity, is important to maintain mitochondrial function. Mitochondria change their function, morphology, and even quantity in response to physiological conditions and stress environments, to keep integrity.^48^ 8-Oxo-dG is a prevalent product of oxidative attacked DNA.^49^ 8-Oxo-dG can be fixed by the base excision repair pathway initiated by OGG1. Livers from OGG1 deletion mice showed up to sixfold higher levels of 8-oxo-dG in nuclear DNA and 20-fold higher in mitochondrial DNA compared with those from wild-type mice.^50^ SIRT3 directly deacetylates OGG1 to protect mitochondrial integrity and prevent apoptotic cell death.^51^ The current data showed that SIRT3 overexpression decreased the acetylation level of OGG1 to reduce 8-oxo-dG content in the *t*-BHP-treated hepatocytes. It suggests SIRT3 has a critical role in repairing mitochondrial DNA damage to protect mitochondrial integrity under oxidative stress.

Mitochondria are highly dynamic organelles, which undergo continuous cycles of fusion and fission to guarantee cellular function. The balance between these two antagonistic processes helps to regulate mitochondrial morphology and maintain mitochondrial network integrity. Alterations of these two opposite processes are observed under both physiologic and pathological conditions. Fission contributes to control mitochondrial quantity and also alleviate the cellular load of damaged mitochondria. Unbalanced fission results in mitochondrial fragmentation. Drp1 is one of the principal proteins participating in mitochondrial fission.^52^ Overexpression of Drp1 promotes increased mitochondrial fission.^53^ We observed mitochondria suffered fragmentation in AML12 hepatocytes exposed to *t*-BHP-induced oxidative stress and SIRT3 overexpression alleviated it. Moreover, SIRT3 overexpression hindered Drp1 mitochondrial translocation through Ku70-Bax-Drp1 axis. Although SIRT3 might be involved in yet unknown fission mechanisms, our results supported that SIRT3 mitigates mitochondrial fragmentation, leading to attenuation of mitochondrial dysfunction and subsequent cell injury.

**Figure 8:**
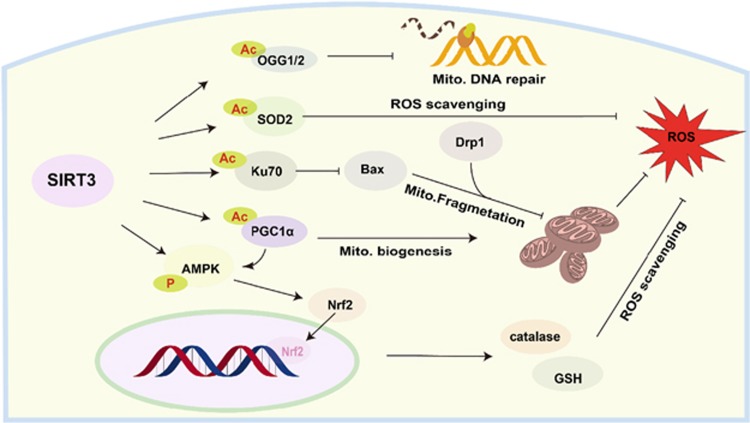
Schematic diagram of the role of SIRT3 in protecting hepatocytes from oxidative injury

In conclusion, we determined SIRT3 level is decreased in oxidative injured hepatocytes *in vitro* and *in vivo*. More importantly, we verified SIRT3 protects hepatocytes against oxidative injury through enhancing anti-oxidative capacity, improving mitochondrial biogenesis, and preventing mitochondrial fragmentation, to maintain mitochondrial morphology and integrity (Figure 8). SIRT3 could be a potential target in the management of hepatic diseases.

## Materials and methods

### Animals

Male C57BL/6 mice (aged 6–8 weeks) were purchased from the animal facility of Faculty of Health Sciences, University of Macau. The mice were fed a regular chow diet (Guangdong Medical Lab Animal Center, Guangzhou, Guangdong, China) and water *ad libitum*. The mice were housed under standard light (i.e., 12/12 h light/dark), temperature (21±2 °C), and relative humidity (60±10%) conditions. The mice were randomly divided into two groups (*n*=10–12 in each group). Acute liver damage was induced by oral administration with 158 mg/kg CCl_4_ (1% (v/v) in olive oil) for the CCl_4_ groups of mice. Control group mice were treated with 10 ml/kg olive oil. After 24 h, the mice were sacrificed under isoflurane anesthesia. Serum was obtained from blood via centrifugation at 3000 × *g* for 20 min. Hepatic samples of the middle of right lobe were fixed in 4% paraformaldehyde immediately for morphological examination, and the remaining liver tissues were snap frozen and stored at −80 °C for other studies. All animal experiments were approved by the Experimental Animal Ethics Committee at the University of Macau.

### Determination of GSH, SOD2, AST, and ALT levels

The levels of GSH and SOD2 in AML12 cells and mouse livers, as well as the levels of AST and ALT in mouse serum, were determined using commercial assay kits (Nanjing Jiancheng, Nanjing, Jiangsu, China) in accordance with the manufacturer’s protocols. Protein concentration was determined by a Pierce BCA Protein Assay Kit (Thermo Fisher, Rockford, IL, USA). GSH and SOD2 levels were normalized by total protein.

### Cell culture

The murine hepatocyte AML12 cell line was purchased from American Type Culture Collection (Rockville, MD, USA). AML12 cells were cultured in DMEM supplemented with 10% FBS and ITS-G (5 mg/ml insulin, 5 mg/l transferrin, and 5 *μ*g/l selenous acid) (Peiyuan, Shanghai, China) in humidified air containing 5% CO_2_ at 37 °C.

### Generation of SIRT3 overexpression cell line

The pcDNA3.1-SIRT3-flag plasmid was generated as previously described.^54^ The pcDNA3.1-SIRT3-flag and pcDNA3.1 plasmids were transfected into AML12 cells or primary hepatocytes using Lipofectamine 2000 (Invitrogen, Carlsbad, CA, USA) in accordance with the manufacturer’s instruction.^55^ In brief, AML12 cells (4 × 10^5^) were seeded in 35 mm plates. After 24 h, the cells were transfected with 10 *μ*g plasmids (pcDNA3.1 or pcDNA3.1-SIRT3-flag) using Lipofectamine 2000 reagent. At 24 h after transfection, 800 *μ*g/ml G418 (Sigma, Santa Clara, CA, USA) was added to select positive cells for 12 days. The medium was changed every other day.

### Cell viability assays

The viability of AML12 cells was determined using MTT. In brief, AML12 cells (1 × 10^4^ cells/well) were seeded into a 96-well plate and then cultured for 16 h. For vector and SIRT3OV cells, 500 *μ*M *t*-BHP or the same volume of DMSO was added. After 12 h, 1 mg/ml MTT solution was added to each well, and the 96-well plates were further incubated for 4 h at 37 °C. Subsequently, 100 *μ*l DMSO was added to each well to solubilize formazan precipitates. Absorbance at 570 nm was measured using a microplate reader (Flexstation 3, Molecular Devices, CA, USA).

The cell viability was also evaluated by a trypan blue exclusion assay. Cells were seeded at a density of 5 × 10^4^ per well in 12-well plates. The number of viable cells were counted by 0.4% trypan blue (Invitrogen) exclusion using a hemocytometer and expressed as a percentage relative to vector control.

### Intracellular ROS detection

Intracellular ROS levels were detected using DCFH-DA (Invitrogen) as previously described.^56^ In brief, AML12 cells (1 × 10^5^) were seeded into each well of a 96-well black multitier plate with a clear bottom and then cultured overnight. The cells were treated as described in cell viability section. After 12 h, the cells were incubated with DCFH-DA (10 *μ*M) at 37 °C in the dark for 15 min. Fluorescence intensity was analyzed through FACSCalibur flow cytometry (BD, Lake Franklin, NJ, USA).

### Immunoblotting

Primary hepatocytes and AML12 cells were lysed with RIPA lysis buffer (Beyotime, Shanghai, China) containing 1% protease inhibitor cocktail and 1% phenylmethane sulfonylfluoride (Sigma). Protein concentration was determined using a BCA Protein Assay Kit. An equal amount of proteins was separated using 5–12% sodium dodecyl sulfate-polyacrylamide gel electrophoresis and then transferred to polyvinylidene fluoride membranes. After blocking with 5% nonfat milk for 2 h at room temperature, the membranes were probed with specific primary antibodies overnight at 4 °C and then probed with corresponding secondary antibodies for 1 h at room temperature. Signals were developed using a SuperSignal West Femto Maximum Sensitivity Substrate kit (Thermo). Then, specific protein bands were visualized using the CheniDoc MP Imaging System. Quantification was performed with Image Lab 5.1 (Bio-Rad, Hercules, CA, USA). The detailed antibodies information was listed in Supplementary Table 1.

### Nucleus isolation

Nuclear proteins were extracted from AML12 cells using a Nuclear Protein Extract Kit (Beyotime). In brief, 2 × 10^6^ cells were re-suspended in 200 *μ*l buffer A and then vortexed for 5 s. After 15 min of incubation on ice, 10 *μ*l buffer B was added and then vortexed for 5 s. The supernatant was discarded after centrifugation at 16 000 *g* for 5 min at 4 °C. The pellet was re-suspended in 50 *μ*l nuclear extraction buffer and then vortexed for 30 s. The nuclei were collected in the supernatant by centrifugation at 16 000 *g* for 5 min at 4 °C.

### Mitochondrial isolation

Mitochondrial fraction was isolated from cells using mitochondrial isolation buffer (pH: 7.5, 210 mM mannitol, 70 mM sucrose, 1 mM EDTA, 10 mM HEPES). In brief, 5 × 10^6^ cells were re-suspended in 1 ml buffer and homogenized for 30–45 seconds on ice using a Dounce Homogenizer at 20 000 rpm/min. Cell slurry was transferred to microfuge tubes and centrifuged at 400 × *g* for 10 mins at 4 °C. The supernatant was transferred to a new tube and centrifuged at 10 000 × *g* for 10 min at 4 °C. The mitochondrial fraction was collected and resuspend in RIPA lysis buffer (Beyotime) containing 1% protease inhibitor cocktail and 1% phenylmethane sulfonylfluoride (Sigma).

### Mitochondrial membrane potential (ΔΨm) assay

ΔΨm was determined by the fluorescent dye Rhodamine123 (R123), a cell-permeable cationic dye that preferentially partitions into the mitochondria based on the highly negative ΔΨm. In brief, AML12 cells were treated as described in the cell viability section. Then, the cells were stained with R123 (10 *μ*M) for 10 min. Subsequently, the cells were washed twice with PBS, trypsinized, and then collected in a 1.5 ml tube. The change in ΔΨm was qualitatively observed on an In Cell Analyzer 2000 (GE Healthcare Life Sciences, Chicago, IL, USA).

### Immunoprecipitation

Cell lysates (3 mg protein) were mixed with the indicated antibody (2 *μ*g) at 4 °C overnight. Subsequently, 20 *μ*l protein A/G-agarose beads (Santa Cruz Biotechnology, Santa Cruz, CA, USA) were added to the cell lysate and incubated on a rotator for 4 h at 4 °C. The beads were washed thrice with PBS and then twice with lysis buffer supplemented with complete mini-protease inhibitor cocktail. Bound proteins were boiled in sample preparation buffer for 5 min.

### Real-time quantitative PCR

Total DNAs were extracted from AML12 cells with TRIzol (Invitrogen). To determine the ratio of mtDNA/nuclear DNA copy number, a quantitative RT-PCR was performed on Mx3005P qPCR System using SYBR green detecting method (Agilent, Santa Clara, CA, USA). Two pairs of primers were used to quantify and confirm relative mtDNA/nuclear DNA ratio: ND1 for mtDNA, H19 for nuclear DNA. Results were confirmed by at least three independent experiments. All of the PCR primers were synthesized by Sigma. The qPCR program is as follows: 95 °C 1 min for 1 circle; 95 °C 15 s, and 60 °C 60 s for 40 cycles. Primers used for the PCR are listed as follows. ND1 forward primer: 5'-AATCGCCATAGCCTTCCTAACAT-3', reverse primer: 5'-GGCGTCTGCAAATGGTTGTAA-3'. H19 forward primer: 5'-GTACCCACCTGTCGTCC-3', reverse primer: 5'-GTCCACGAGACCAATGACTG-3'.

### Isolation of primary hepatocytes

Primary hepatocytes were isolated using the two-step Percoll gradient method as previously described.^57^ The liver was perfused with Ca^2+^ and Mg^2+^-free Hank’s buffered salt solution (HBSS, Gibco, Grand Island, NY, USA) containing EGTA (2.5 mM) and then digested with collagenase buffer (0.5 mg/ml collagenase type IV (Roche, Basel, Switzerland), 66.7 mM NaCl, 6.7 mM KCl, 50 mM HEPES, and 4.8 mM CaCl_2_). Digested liver was dissected and then gently teased into small pieces with forceps. Liver slurry was filtered through a 100 *μ*m nylon cell strainer (BD). After spin down at 1200 × *g* for 5 min, the cell pellet was re-suspended with HBSS. The cell suspension was centrifuged at 400 × *g* for 3 min, and the pellet was re-suspended in 25% Percoll and then centrifuged at 550 × *g* for 5 min with the brake option off. The pellet was washed once with DMEM supplemented with 10% FBS. Then, the cells were seeded into collagen precoated 100 mm plates. After 24 h, the adherent cells were collected for the following experiments.

### RNA Interference

The shRNA-targeting SIRT3 (mouse, sc-61556) and scrambled RNA (mouse, sc-108060), and shRNA transfection reagent (mouse, sc-108061) were purchased from Santa Cruz Biotechnology. AML12 cells were transfected with 2 *μ*g shRNA for 6 h according to the manufacturer's protocol. Cells were switched to fresh medium and incubated for an additional 24 h. Then, cells were selected with 2 *μ*g/ml puromycin for 6 days, and then 4 *μ*g/ml puromycin for 6 days. Thereafter, cells were pooled together for further experiments.

### Statistical analysis

Data were analyzed using GraphPad Prism-6. All experimental data were expressed as mean±S.D., and each experiment was performed a minimum of three times. *P*<0.05 was considered to be statistically significant.

## Publisher’s Note

Springer Nature remains neutral with regard to jurisdictional claims in published maps and institutional affiliations.
