# Supplementary material for: SIRT3 protects hepatocytes from oxidative injury by enhancing ROS scavenging and mitochondrial integrity
Source: Cell Death Dis. 2017 Oct 26;8(10):e3158–. doi: 10.1038/cddis.2017.564 (PMC5680927; doi:10.1038/cddis.2017.564)
Supplement: Supplementary Figures [file cddis2017564x1.pdf]

Supplementary Figure S1

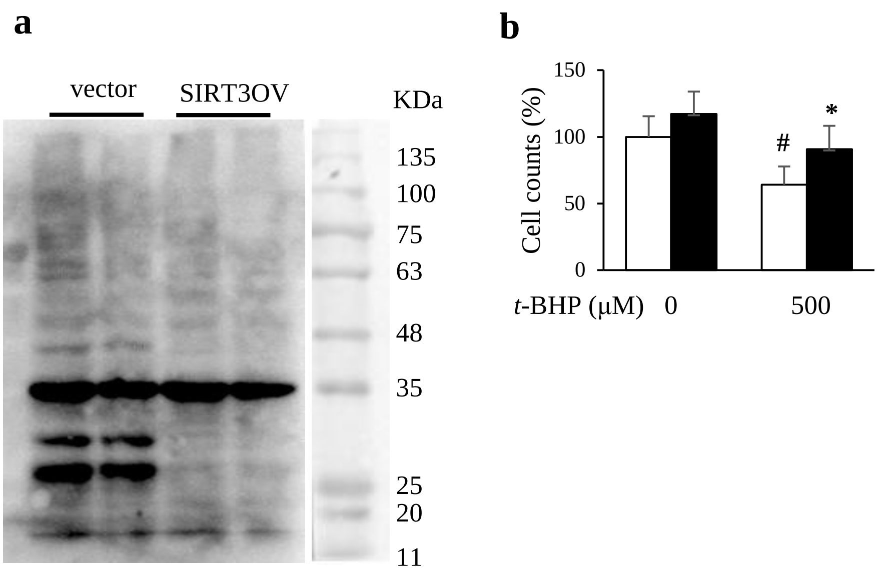

Supplementary Figure S1 (**a**) SIRT3 overexpression decreased acetylation level of total protein in *t*-BHP-treated AML12 cells. (**b**) Cell viability was determined by trypan blue dye exclusion. The number of viable cells were counted by 0.4% trypan blue stain exclusion using a hemocytometer and expressed as a percentage relative to control cells. $*p < 0.05$, and SIRTOV vs. vector cells, and $\#p < 0.05$ *t*-BHP-treated vs. ctrl cells.

Supplementary Figure S2

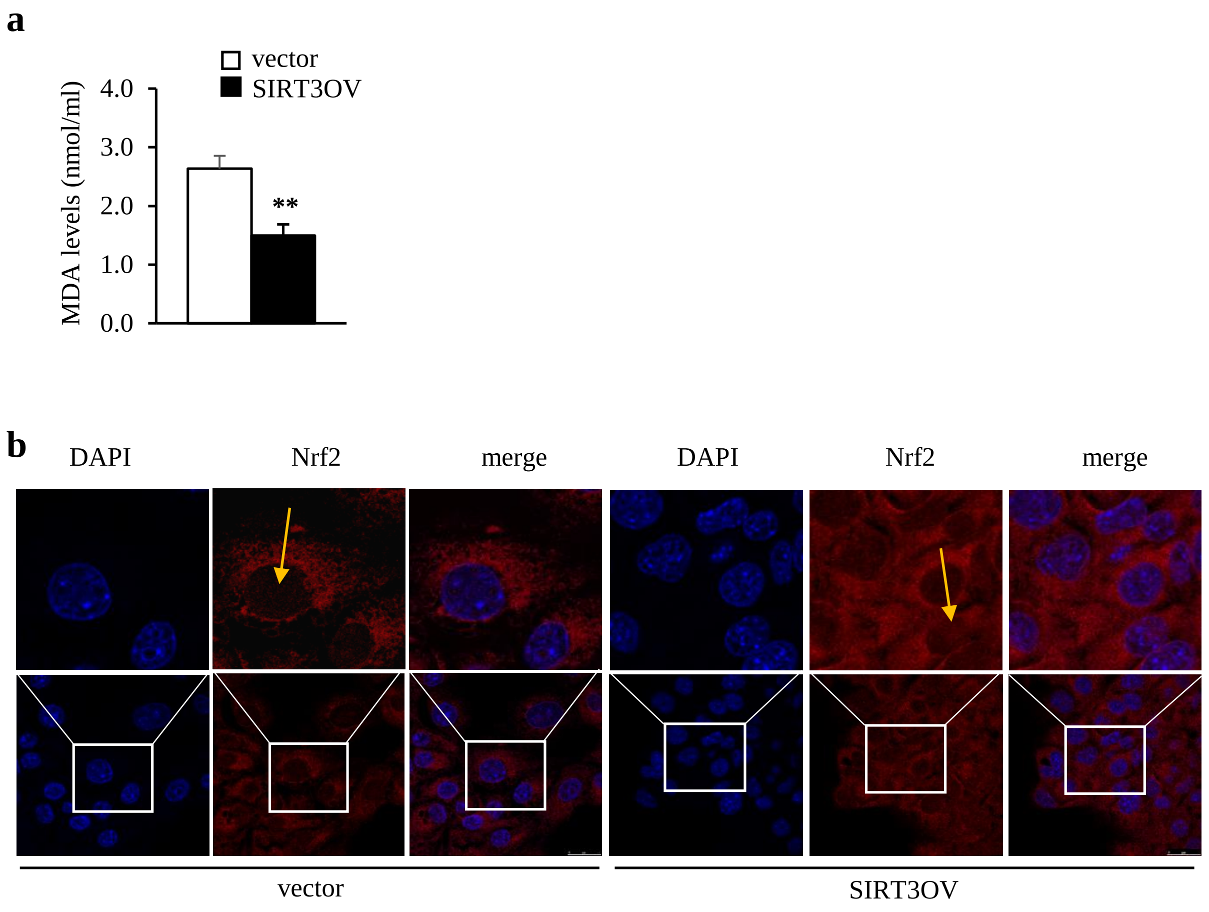

Supplementary Figure S2 (**a**) Intracellular MDA(malondialdehyde) level. (**b**) Confocal immunofluorescence image of total and nuclear Nrf2 protein. Nrf2 and nuclei were stained by Texas Red and DAPI, respectively. Scale bar, 10 μm. **p < 0.01, and SIRT3OV vs. vector cells.

Supplementary Figure S3

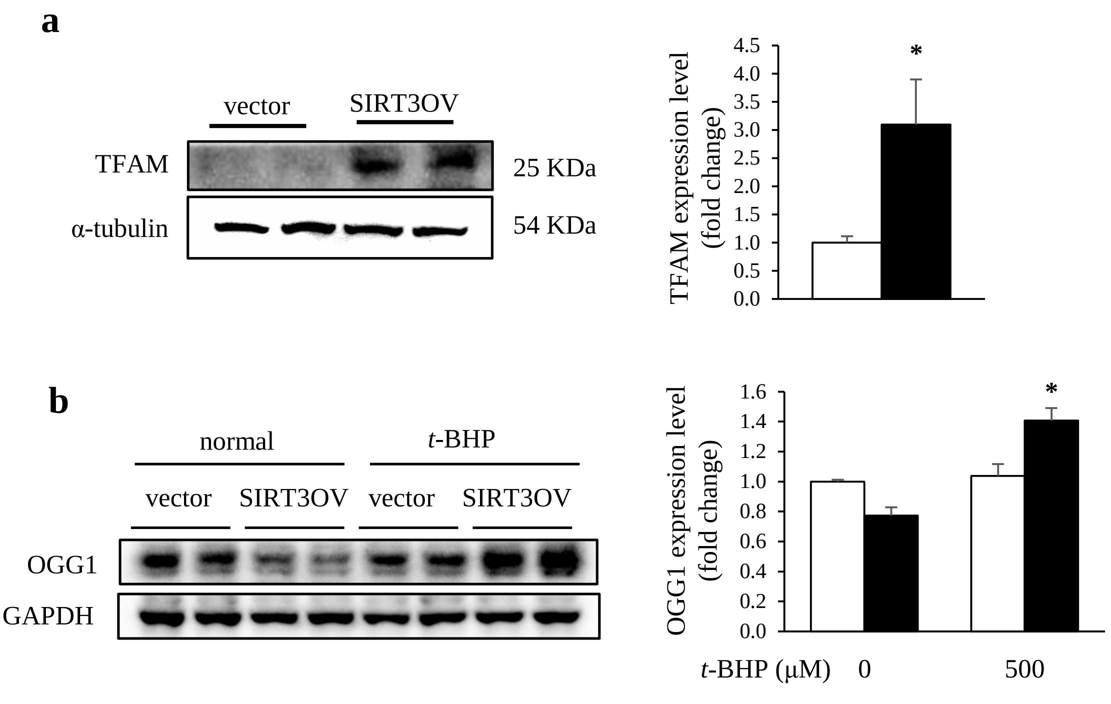

Supplementary Figure S3 (**a**) TFAM protein level in vector and SIRT3 overexpressed AML12 cells treated with 500 μM *t*-BHP for 12h. (**b**) OGG1 expression in the vector and SIRT3OV cells under normal and *t*-BHP treated conditions. *$p < 0.05$, and SIRT3OV vs. vector cells.

Supplementary Table 1 The detailed information of antibodies used in immunoblotting.

Name	Cat.No.	Company
p-AMPK	SC-33524	Santa Cruz
AMPK	SC-25792	Santa Cruz
PGC1α	AB54481	Abcam
Tom20	SC-17764	Santa Cruz
Drp1	#8570	CST
MFN1	SC-50330	Santa Cruz
Nrf2	#12721	CST
KEAP1	#8047	CST
OPA1	SC-30573	Santa Cruz
α-tubulin	SC-8035	Santa Cruz
GAPDH	SC-25778	CST
Ku70	SC-135963	Santa Cruz
Bax	SC-526	Santa Cruz
SOD2	24127-1-AP	Proteintech Group
histone 3	SC-10809	Santa Cruz
OGG1	SC-376935	Santa Cruz
catalase	SC-50508	Santa Cruz
β-actin	SC-1616	Santa Cruz
SIRT3	#5490	CST
Acetylated-Lysine	#9681	CST
Tom20	#42406	CST
MFF	17090-1-AP	Proteintech Group
MID49	16413-1-AP	Proteintech Group
Tom40	18409-1-AP	Proteintech Group
Sam50	20824-1-AP	Proteintech Group
Donkey anti-goat IgG HRP	SC-2020	CST
anti-rabbit IgG HRP	#7074	CST
anti-mouse IgG HRP	#7076	CST
